# Close Collaboration of Two Transplantation Research Centers in Iran

**Published:** 2013-08-01

**Authors:** Karim Vessal

From April 2013, the *IJOTM* has extended its activities in publishing articles on tissue banking. It is a great pleasure to witness expansion of the journal activities in this new dimension that heralds a bright future through interactive reinforcement of the country’s two major transplantation units by sharing mutual experience to further the regional goals of the MESOT. On account of the prevailing sanctions on the overall growth of the country’s scientific output, policies to optimize funds and efforts are most welcome.

The decision to create closer ties and enhance existing collaboration in research and publication between the two centers, was reached in the latest session of MESOT between Dr. Malek-Hosseini from Transplantation Research Center, Shiraz University of Medical Sciences and Dr. Mahdavi-Mazdeh from Tissue Bank, Tehran University of Medical Sciences. We anticipate fruition of this merger in our scientific and health care endeavors and will brief our interested audience of the outcome of our policies in due time.

